# Low-Power and Wireless Communication Research on Underground Displacement Three-Dimensional Monitoring System

**DOI:** 10.3390/s24051592

**Published:** 2024-02-29

**Authors:** Nanying Shentu, Xianyang Zhang, Qing Li, Renyuan Tong, Guohua Qiu

**Affiliations:** 1 College of Mechanical and Electrical Engineering, China Jiliang University, Hangzhou 310018, China; stnying@cjlu.edu.cn (N.S.); zxyang0221@163.com (X.Z.); tongrenyuan@126.com (R.T.); 2 College of Information Engineering, China Jiliang University, Hangzhou 310018, China; qghfr@163.com

**Keywords:** underground displacement, three-dimensional measurement, Bluetooth wireless transmission technology, low power consumption, dynamic power management

## Abstract

Underground displacement monitoring is a crucial means of preventing geological disasters. Compared to existing one-dimensional methods (measuring only horizontal or vertical displacement), the underground displacement three-dimensional measurement method and monitoring system proposed by the author’s research team can more accurately reflect the internal movement of rock and soil mass, thereby improving the timeliness and accuracy of geological disaster prediction. To ensure the reliability and long-term operation of the underground displacement three-dimensional monitoring system, this article further introduces low-power design theory and Bluetooth wireless transmission technology into the system. By optimizing the power consumption of each sensing unit, the current during the sleep period of a single sensing unit is reduced to only 0.09 mA. Dynamic power management technology is employed to minimize power consumption during each detection cycle. By using Bluetooth wireless transmission technology, the original wired communication of the system is upgraded to a relay-type wireless network communication, effectively solving the problem of the entire sensing array’s operation being affected when a single sensing unit is damaged. These optimized designs not only maintain monitoring accuracy (horizontal and vertical displacement errors not exceeding 1 mm) but also enable the monitoring system to operate stably for an extended period under harsh weather conditions.

## 1. Introduction

Geological disasters mainly refer to collapses (i.e., dangerous rock masses), landslides, mudslides, karst ground collapses, and ground fissures, with a wide variety of types and serious hazards. The occurrence of geological disasters not only seriously threatens people’s life safety but also endangers economic development and the geographical environment [1]. Underground displacement monitoring is an important method of preventing and studying geological disasters. It can dynamically monitor geological parameters such as displacement value, direction, and rate inside rock and soil mass, issue timely warnings, and facilitate the organization of crowd evacuation, ensuring the safety of people’s lives and property [2,3,4]. However, since the monitoring environment for underground displacement is located inside the rock and soil mass, many methods are difficult to use for underground displacement monitoring. At present, domestic and foreign methods for underground displacement monitoring mainly include borehole inclinometers, optical fiber sensing technology, and array displacement meters [5,6,7,8,9,10]. However, these methods cannot achieve the monitoring of underground three-dimensional displacement of rock and soil mass and cannot truly reflect the evolution process of underground rock and soil mass. In recent years, our research group has integrated multiple magnetoelectric sensing mechanisms such as electromagnetism, mutual inductance, and magnetoresistance, combined with dual mutual inductance equivalent modeling, a flexible sensing array, and integrated sensing technology, to propose a new type of underground displacement remote monitoring system [11,12], which can automatically, in real-time, and accurately monitor the underground three-dimensional displacement of rock and soil mass. Through the study of the above literature, the advantages and disadvantages of existing underground displacement detection methods are shown in Table 1.

The system is usually installed in high mountains or landslide-prone zones. Due to the steep terrain, narrow and rugged roads, and lack of power supply facilities, it can only be powered by batteries or solar panels. Due to the high power consumption of the entire system, when encountering cloudy or rainy weather, the solar panels will stop working, and the batteries can only provide power for less than a day. Therefore, it is necessary to replace the batteries in a timely manner. However, the installation site environment of the monitoring system is complex and changeable. Strong winds and rainstorms often attack, and weeds are overgrown and muddy. These factors make battery replacement difficult and require several people to work together. At the same time, these factors increase the complexity and risk of the work and bring huge troubles and challenges to the installation personnel. Therefore, it is necessary to design the system with low power consumption. In modern electronic devices, low-power design has become the mainstream design concept. Not only can it extend the service life of the device and save energy costs, but it can also better adapt to the requirements of battery powered, wireless transmission, and other application scenarios. At present, low-power design methods can be divided into hardware low-power design and software low-power design. Generally, software and hardware are used in conjunction for the low-power design of the system [13,14]. Ren, Z. et al. [15] proposed a hierarchical scheme for adaptive dynamic power management, optimizing battery life by transitioning the device to a low-power mode during periods of reduced service demand. Martínez-Suárez, F. et al. [16] created an energy-efficient, long-term ambulatory ECG (electrocardiogram) monitor. They focused on optimizing the hardware circuitry, incorporating low-power components and processors, and implementing an efficient hibernation mode. Sun, Z. et al. [17] suggested employing stacked multiple low-power circuits as a substitute for voltage converters in conventional designs. This approach saves one or more voltage converters and eliminates power losses typically linked to converters. Qian, F. W. et al. [18] introduced a design methodology for low-power CMOS integrated circuits, addressing both dynamic and static power sources. The approach involves load capacitance reduction, the utilization of dual threshold voltages, and minimizing clock jumps. Jahanirad, H. [19] proposed an energy-efficient architecture for static random access memory (SRAM)-based FPGAs. The architecture defines each module with two modes: active and sleep. By flexibly switching between these modes, the FPGA can effectively reduce leakage power consumption. Sidibe, A. et al. [20] devised two distinct power management units. These units leverage low-power components, effectively overseeing and storing the required energy in storage capacitors. This strategy reduces the overall power consumption of the system. Sun Xiyuan [21] employed the concept of parallel processing to disassemble a single functional module into several identical sub-modules. This parallelization approach aims to minimize circuit power consumption while preserving circuit performance. Tuncel, Y. et al. [22] introduced a system-level framework integrating domain-specific knowledge with low-power design techniques. This framework aims to reduce the energy consumption of wearable health and activity monitoring applications.

The original system [11,12] used an RS485 bus to connect the ground management terminal and hundreds of sensing units for communication. If the 485 communication module of any sensing unit has failed for some reason, it will cause other sensing units to be unable to continue 485 communication. In addition, the sensing units are deeply buried underground, so it is almost impossible to replace the sensing units. Therefore, it is necessary to switch from wired communication to wireless communication. Wireless communication technology can be divided into short-distance wireless communication technology and long-distance communication technology. Short-distance communication technology includes infraredcommunicat, ZigBee, WiFi (Wireless Fidelity), BLE (Bluetooth Low Energy), etc. Long-distance communication technology includes LoRa (Long Range Radio) technology, NB-IOT (Narrow Band Internet of Things) technology, etc. [23,24,25,26,27,28,29,30,31,32]. In order to meet the special needs of complex geotechnical environments deep underground, it is necessary to study wireless communication technology and the network topology structure suitable for an underground displacement three-dimensional monitoring system. In the initial state, considering the structure of the sensing units, the relative displacement between the circuit boards of the two sensing units is generally around 10 cm [12]. At the same time, based on the author’s extensive previous research, for the majority of geological disasters, the relative displacement between any two sensing units is not expected to exceed 15 cm. Moreover, short-range wireless communication technology can achieve communication distances of over 10 m. Therefore, it is only necessary to consider short-range wireless communication technology. After consulting a large amount of information, this article summarizes the main parameters and advantages and disadvantages of four short-distance wireless communication technologies, as shown in Table 2.

According to Table 2, it is evident that all four short-range communication methods meet the communication distance requirements of the monitoring system. Nevertheless, as the monitoring system is buried underground, the installation process involves drilling holes in the designated area, deploying sensing units, and pouring a substantial amount of water into the borehole to cool the drill bit. After completing the mountain drilling, the borehole becomes filled with water, and the soil inside gradually settles under the influence of soil pressure, leading to water overflow. At this stage, the entire sensing array becomes immersed in water. When the water inside the borehole completely overflows, the sensing array is then in a mixture of mud and water. In this situation, both infrared communication technology and Zigbee technology face challenges due to object obstruction, resulting in limitations on data transmission. In addition, the power consumption of WiFi wireless communication is usually dozens of times that of Bluetooth wireless communication. While ensuring transmission efficiency, in order to pursue lower power consumption, Bluetooth wireless communication technology was ultimately chosen.

Contributions:

- For the first time, low-power design theory, methods, and technologies have been applied to underground displacement three-dimensional monitoring systems. In hardware, low-power components are selected, and the sensing unit circuit is modularized, adopting controllable power management. In software, multiple working modes are divided and combined with controllable power management modules to arrange the working time of each functional module of the sensing units reasonably, achieving dynamic power management of the sensing unit and sensing array.
- A power consumption model has been established for the newly designed monitoring system, providing a reference for the subsequent selection and installation of sensing units and power supplies in the field.
- A significant advancement is the integration of wireless communication technology into the underground displacement three-dimensional monitoring system, replacing the previous wired communication with Bluetooth wireless communication. This solution addresses the issue of the entire sensing array becoming unusable due to a single sensing unit failure.

This article is organized as follows: Section 2 introduces the overall structure of the underground displacement three-dimensional monitoring system and the basic working principles of the sensing units. Section 3 describes the hardware design of the sensing units and ground management terminal and then optimizes the hardware of each functional module of the sensing units. Section 4 designs a relay wireless communication network suitable for the monitoring system and then designs the corresponding software workflow to achieve the functions of the sensing units in different working modes. Section 5 establishes the power consumption model of the sensing array and then conducts power consumption testing on the sensing array composed of 10 sensing units, verifying the power consumption model based on the test results. The conclusions are in Section 6.

## 2. Overall Structural Design and Working Principle

### 2.1. System Structure Design

**Figure 1:**
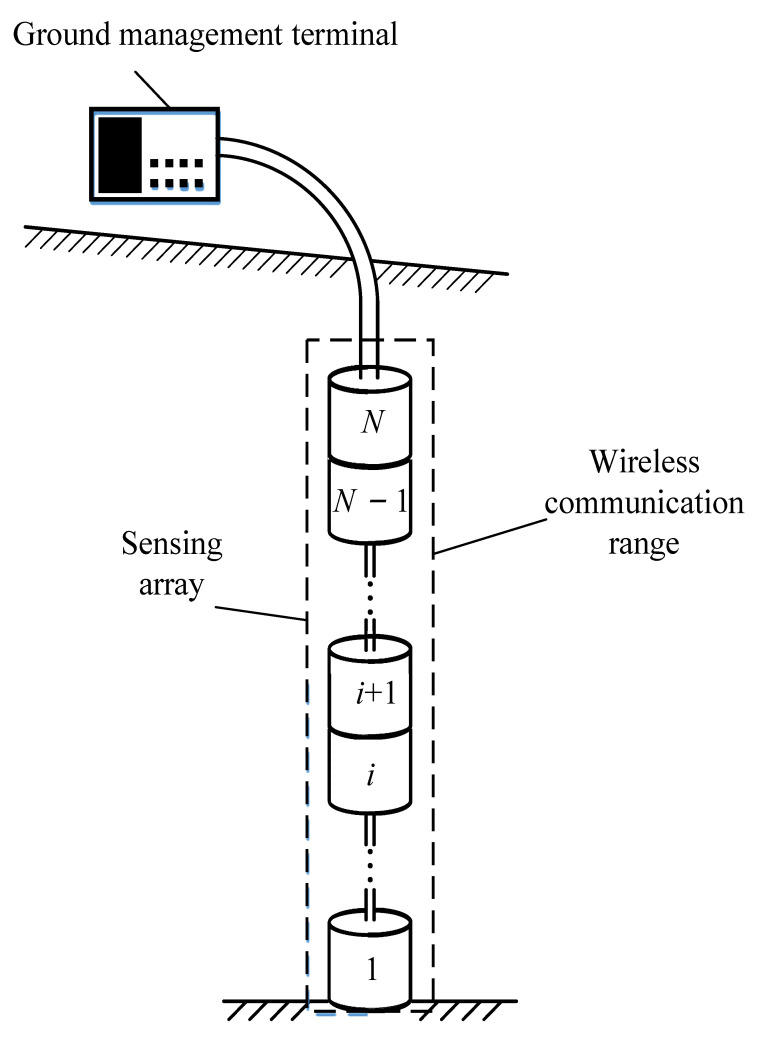
Underground displacement three-dimensional monitoring system.

The underground displacement three-dimensional monitoring system includes a ground management terminal and flexible sensing array made of multiple sensing units connected in series through power lines, as shown in Figure 1. Among them, the ground management terminal mainly performs the functions of waking up the sensing array to work, saving the data collected by the sensing array, and sending them to the AliCloud server. The No. *N* sensing unit in the sensing array is connected to the ground management terminal through a power line and RS485 communication line, transmitting the data information collected by the sensing array to the ground management terminal for processing. The sensing units (No. 1 ~ No. *N*) in the sensing array transmit data by means of wireless communication. In the sensing array, except for sensing units No. 1 and No. *N*, all the other sensing units need to act as both the excitation end to generate sinusoidal signals and the measurement end to collect the mutual inductance voltage generated by the mutual inductance phenomenon, and the measurement sequence of the sensing array is from bottom to top. When the monitoring system is actually used, the number of sensing units is not fixed and can be adjusted according to actual needs. Taking a mid-level landslide as an example, where the thickness of the landslide body typically ranges from 6 m to 20 m, the number of sensing units may reach the hundreds. And each sensing unit has the same structure and circuit. The power consumption of the sensing array accounts for over 90% of the entire system. Therefore, this article mainly aims to reduce the power consumption of the entire system by reducing the power consumption of each sensing unit.

### 2.2. Working Principles of the Sensors

**Figure 2:**
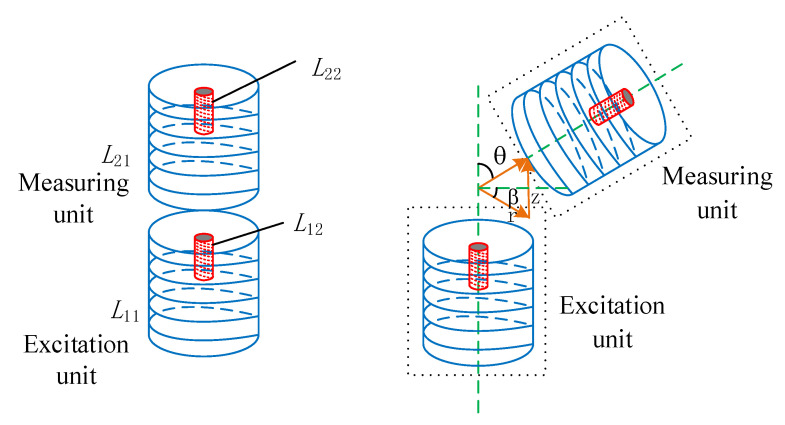
Principle diagram of dual mutual inductance voltage measurement.

In two adjacent coils, when the current in one coil changes, an induced electromotive force is generated in the adjacent coil, which is called the mutual inductance phenomenon. The principle of dual mutual inductance measurement proposed by our research group [11,12] is based on the application of the mutual inductance phenomenon. Figure 2 shows the principle diagram of dual mutual inductance voltage measurement.

Both the excitation end and the measurement end contain an air-core coil and a magnetic core coil. For convenience, the air-core coil and magnetic core coil at the excitation end are labeled as *L*_11_ and *L*_12_, respectively, and the air-core coil and magnetic core coil at the measurement end as *L*_21_ and *L*_22_, respectively. When a sine signal with constant frequency and stable amplitude is input into *L*_11_, a stable alternating magnetic field is generated, causing an induced voltage to be generated at both ends of *L*_21_. This induced voltage is rectified into a DC voltage through a collection circuit and referred to as type I mutual inductance voltage *U*_1_. Similarly, the voltage collected by exciting coil *L*_21_ with coil *L*_12_ is called type II mutual inductance voltage *U*_2_.

The attitude detection module integrated within the sensing unit can obtain the azimuth angle (*β*) of the measuring end itself and the relative tilt angle (*θ*) between the excitation end and the measurement end in real time. Next, through calibrating the variation data of the double mutual-inductance voltage at the measurement end with respect to the changes of relative horizontal displacement *r*_i_ and relative vertical displacement *z*_i_ at different tilt angles *θ*, a measurement model can be established to depict the relationship between the displacements to be measured (including the relative horizontal displacement *r*_i_ and relative vertical displacement *z*_i_) and the measured parameters, including the dual mutual inductance voltages *U*_1_ and *U*_2_, the tilt angle *θ*, and the azimuth *β*. The model is shown in Equation (1).

(1) $$\left\{\begin{array}{c} r_{i}=f_{1}(U_{1},U_{2},\theta ,\beta )\\ z_{i}=f_{2}(U_{1},U_{2},\theta ,\beta ) \end{array}\right.$$

Applying this principle to underground displacement measurement, we only need to vertically place multiple sensing units in the rock and soil mass and reflect the overall horizontal displacement *R* and vertical displacement *Z* inside the borehole by overlaying the relative horizontal displacement *r*_i_ and relative vertical displacement *z*_i_ between any two adjacent sensing units, as shown in Equation (2).

(2) $$\left\{\begin{array}{l} R=\sum_{i=1}^{N-1}r_{i}\\ Z=\sum_{i=1}^{N-1}z_{i} \end{array}\right.$$

## 3. Hardware Optimization Design

### 3.1. Hardware Block Diagram of the Sensing Unit

**Figure 3:**
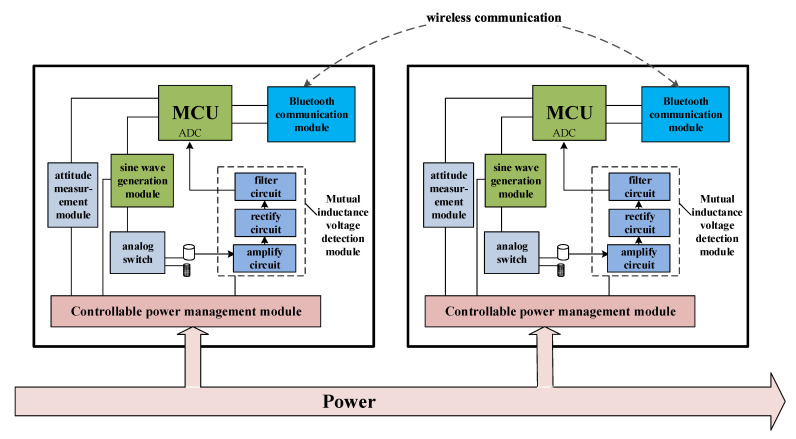
Hardware diagram of the sensing unit.

Figure 3 shows the hardware diagram of the sensing unit, which mainly includes seven functional modules. They are the microprocessor (MCU), sine wave generation module, mutual inductance voltage detection module, attitude detection module, analog switch, Bluetooth communication module, and controllable power management module. When the sensing unit serves as the excitation end, the microprocessor controls the sine wave generation circuit to generate a sine voltage signal with a fixed frequency and amplitude, which is amplified and input into the air-core coil or magnetic core coil through an analog switch. When the sensing unit serves as the measurement end, its mutual inductance voltage detection module amplifies, rectifies, and filters the type I and type II mutual inductance AC voltages obtained by electromagnetic coupling of the air-core coil, converting them from AC signals to DC signals. Then, they can be input to the microprocessor for ADC (analog-to-digital converter) conversion and data analysis. The attitude measurement module collects the real-time spatial attitude data of the sensing unit and transmits them back to the microprocessor (MCU). Through the built-in spatial attitude analysis model, the tilt angle (*θ*) and azimuth (*β*) of each sensing unit are accurately calculated. Two sensing units communicate wirelessly through Bluetooth communication modules to achieve data exchange.

### 3.2. Hardware Design of the Ground Management Terminal

**Figure 4:**
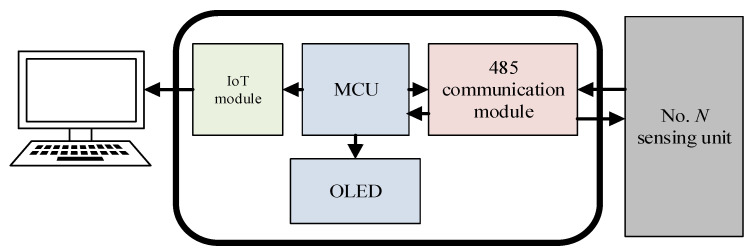
Hardware diagram of the ground management terminal.

The hardware diagram of the ground management terminal is shown in Figure 4. The circuit of the ground management terminal can be divided into four parts, which are the microprocessor (MCU), 485 communication module, OLED (Organic Light-Emitting Diode) module, and IoT (Internet of Things) module. In the monitoring system, due to the sensing array being buried deep underground, it is usually hundreds of meters away from the ground management terminal. To ensure that the data collected by the sensing array can be accurately transmitted to the ground management terminal, a 485 module communication connection is used between the ground management terminals and the No. *N* sensing unit. When the entire monitoring system starts working, the microprocessor sends a wake-up command to the No. *N* sensing unit through the 485 module. After the sensing array collects data, all the data are sent to the microprocessor. The microprocessor analyzes and processes the collected data to calculate the displacement information underground, and then uploads the relevant information to the PC through the IoT module and displays them on the OLED screen.

### 3.3. Optimization of Power Consumption

In order to reduce the power consumption of the system and extend the battery life, this article has carried out the hardware low-power optimization design for the main functional modules of the sensing unit of the monitoring system.

#### 3.3.1. Optimization Design of the Microprocessor and Sine Wave Generation Module

This article uses a low-power chip (STM32L151C8T6 (STMicroelectronics, Geneva, Switzerland)) to replace the original system’s microprocessor. This chip has multiple working modes and can be flexibly switched. As shown in Table 3, there is a significant difference in the operating current of different operating modes of the chip. Therefore, when there is no measurement task, putting the chip into standby mode can greatly save power consumption. In order to reduce the design of peripheral circuits, the sine wave generation module uses the built-in DAC (Digital to Analog Convertor) of the microprocessor to generate sine waves, which are transported by DMA (Direct Memory Access) without passing through the CPU, reducing the burden on the microprocessor core. Meanwhile, this design method replaces the DDS (Direct Digital Frequency Synthesis) module of the original circuit, optimizes the circuit structure, and reduces the power consumption of the sine wave generation module.

#### 3.3.2. Optimization Design of the Attitude Detection Module

The attitude detection module has the highest power consumption proportion in the sensing unit, which can reach over 45%. This module has been in an open state in the original system. But this module does not have to work at all times. Therefore, a switch circuit is added to this module, combined with a controllable power management module, turning it on when attitude information needs to be collected and off when not needed, reducing unnecessary energy loss.

#### 3.3.3. Optimization Design of the Mutual Inductance Voltage Detection Module

The mutual inductance voltage detection module in the original system adopts an integrated module for mutual inductance voltage collection. This article uses low-power operational amplifiers to build amplification and filtering circuits. A rectifier bridge is constructed using two NMOS transistors and two PMOS transistors. The mutual inductance voltage is filtered into a stable DC voltage through amplification, rectification, and filtering, and the microprocessor’s built-in ADC is used for acquisition. Compared to the integrated module of the original system, the present mutual inductance voltage detection module composed of operational amplifiers and discrete components not only ensures the rectification effect but also reduces the overall power consumption of the mutual inductance voltage detection module.

#### 3.3.4. Optimization Design of the Communication Module

The communication module adopts a low-power Bluetooth wireless communication module, E104BT-02, which has an ultra-low power sleep function and a minimum operating current as low as 2 μA when broadcasting is turned on. This module supports various wake-up methods, such as pin wake-up and serial port wake-up. Using the module instead of the original RS485 wired communication module in the monitoring system not only solves the communication defects mentioned in the Introduction, but also reduces the working current of the communication module from the original 5 mA to the microampere level, greatly reducing the power consumption of the communication module.

#### 3.3.5. Controllable Power Management Module

The entire sensing unit circuit in the original system uses a single power supply to power all modules. However, the working times of each module in the sensing unit are different, and the working order varies. This will cause unnecessary power consumption in the entire sensing unit circuit. Therefore, this article summarizes the main chips and modules used in each functional module, as well as their main parameters, as shown in Table 4. According to the power supply voltage range of each module, we apply two power modules, 5 V and 3.3 V, to supply power to the microprocessor, sine wave generation module, attitude detection module, and mutual inductance voltage detection module, respectively. The power module is combined with an analog switch, dividing each module into independent individuals without interfering with each other, to achieve control of the power supply for each module. Finally, in collaboration with the software design introduced later, the working timing of each module is arranged to minimize the power consumption of the entire system within one cycle.

## 4. Software Optimization Design

The optimization design of the software is mainly achieved through dynamic power control technology. This article combines the working principle of sensors to design the working timing of each functional module in the sensing unit and works with the controllable power management module to control the modules that need to work and execute corresponding functions. And the modules that do not need to work enter sleep mode, reducing unnecessary energy loss. The wired communication between sensing units in the underground displacement three-dimensional monitoring system is upgraded to Bluetooth wireless communication; therefore, a relay wireless communication network suitable for this monitoring system is designed in this article. In summary, this chapter will introduce the software optimization design of the monitoring system in two parts: the design of the relay wireless communication network and the design of the software workflow.

### 4.1. Design of the Relay Wireless Communication Network

Communication among sensing units is achieved by the Bluetooth communication module, and the BLE (Bluetooth Low Energy) network topology can be divided into two types: star topology and broadcast group topology [33,34]. The characteristic of a star topology is a one-to-many data communication method, which means that a host can connect multiple slaves, and the information exchange between the host and slaves will not affect the information transmission of other slaves. The main characteristic of the topology structure of a broadcasting group is the point-to-point data communication mode. When a scanning device establishes a connection with a broadcasting device, other broadcasting devices will not be able to obtain broadcasting data from the broadcasting device. The topological structure of broadcast groups is generally suitable for simple and non-networked application scenarios. The monitoring system needs to achieve point-to-point communication between the sensing unit and another sensing unit, and the sensing unit needs to be able to flexibly switch between host and slave modes and communication objects. Therefore, based on the topology of the broadcasting group, this system has redesigned the communication network of the monitoring system.

**Figure 5:**
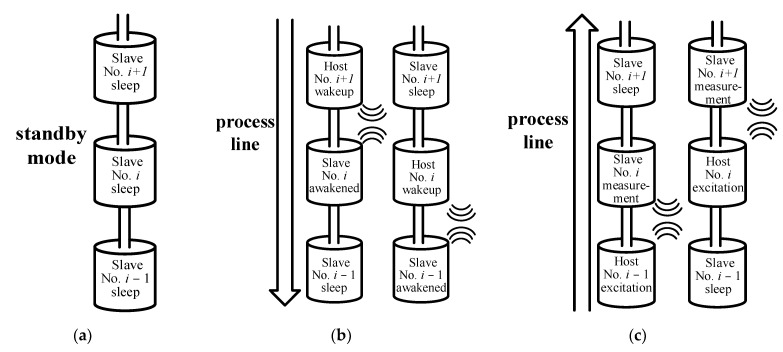
Relay type wireless communication network. (**a**) Standby mode; (**b**) top-to-bottom wake-up mode; (**c**) bottom-to-top communication mode.

When the monitoring system is not conducting measurement work, the entire sensing array is in standby mode, and each sensing unit is in sleep mode. When the system starts measuring, it is necessary to first know how many sensing units in the sensing array can work normally from top to bottom under the normal power supply. At this time, the entire sensing array is in top-to-bottom wake-up mode. When the sensing unit at the bottom of the sensing array is awakened, the monitoring system begins to collect data, and the sensing array enters bottom-to-top communication mode. The bottom sensing unit starts to act as the excitation end, stimulating the sensing unit above it to perform measurement work. In summary, this article designs a relay-type wireless communication network for the monitoring system, as shown in Figure 5.

In standby mode, all sensing units are in slave sleep mode, as shown in Figure 5a. When the monitoring system starts measuring, it enters top-to-bottom wake-up mode, and the workflow of the sensing array is shown in Figure 5b. For example, assuming the No. *i* + 1 sensing unit serves as the topmost sensing unit of the sensing array, the No. *i* + 1 sensing unit is awakened by the ground management terminal and configured as the host. The No. *i* + 1 sensing unit serves as the host to wake up the No. *i* sensing unit, and then the No. *i* sensing unit wakes up the No. *i* − 1 sensing unit, relaying from top to bottom, until the sensing unit that can work normally at the bottom is awakened.

When the sensing unit that can work normally at the bottom is awakened, the sensing array enters bottom-to-top communication mode. For example, when the No. *i* sensing unit can wake up the No. *i* − 1 sensing unit, but the No. *i* − 1 sensing unit cannot wake up the No. *i* − 2 sensing unit below, the No. *i* − 1 sensing unit serves as the host and excitation end and wakes up the No. *i* sensing unit as the measurement end for operation. After this measurement is completed, the No. *i* − 1 sensing unit enters standby mode, and the No. *i* sensing unit serves as the host and excitation end to wake up the No. *i* + 1 sensing unit as the measurement end for operation. The above wake-up and measurement work is carried out sequentially from bottom to top. This arrangement ensures that only two sensing units are in operation during data transmission, while the rest are in sleep mode, greatly saving power consumption. After completing a complete relay transmission, the top sensing unit transmits the data from the entire sensing array to the ground management terminal for processing. 

### 4.2. Software Workflow Design

**Figure 6:**
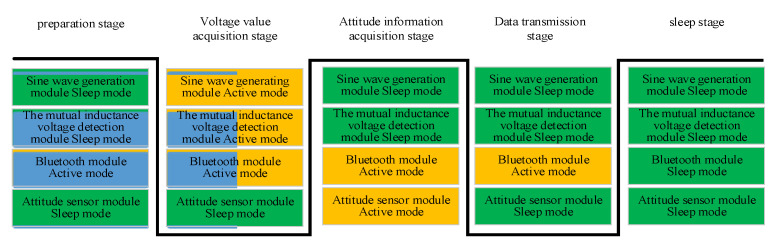
Block diagram of the working modes of each module at different stages.

Based on the introduction of the working principle of sensors in the previous text, the workflow of a sensing unit within a cycle can be divided into five stages: preparation stage, voltage value acquisition stage, attitude information acquisition stage, data transmission stage, and sleep stage. At this time, dynamic power management technology can be used to reasonably arrange the working status of each functional module. Figure 6 shows the working status of the main modules of the sensing unit circuit in each stage.

During the preparation phase, except for the Bluetooth module being in active mode, all other modules are in sleep mode. After the Bluetooth module receives the command, the sensing unit first enters the voltage data acquisition stage. The sine wave generation module of the sensing unit at the excitation end is awakened, and the mutual inductance voltage detection module of the sensing unit at the measurement end is awakened. After the acquisition of voltage data is completed, the sine wave generation module and the mutual inductance voltage detection module return to sleep mode. The sensing unit enters the attitude information acquisition stage, and the attitude detection module enters the active mode. After the acquisition of attitude information is completed, it enters the data transmission stage. At this time, the Bluetooth module sends the voltage value and attitude information to the host. Afterwards, the entire sensing unit enters standby mode, and all modules enter sleep mode.

Combining Figure 5 and Figure 6, it can be seen that the software control of the monitoring system is complex and variable, including many different functions and operations involving the coordination of multiple modules and components. In order to ensure the reliability and maintainability of the program, this article designs a functional code communication protocol suitable for this monitoring system based on the Modbus protocol. The specific format is shown in Table 5. In order to distinguish whether the sensing unit acts as a host or a slave during operation, the first digit of the data packet sent by the host is defined as 0xAA, and the first digit of the data packet sent by the slave is defined as 0xFF. Meanwhile, this article designs different functional codes for each function to implement specific functions, as shown in Table 6. Finally, an error rate of only 2.33 × 10^−10^ was added to CRC32 (Cyclic Redundancy Check 32-bit) verification to ensure the accuracy of transmitted data.

**Figure 7:**
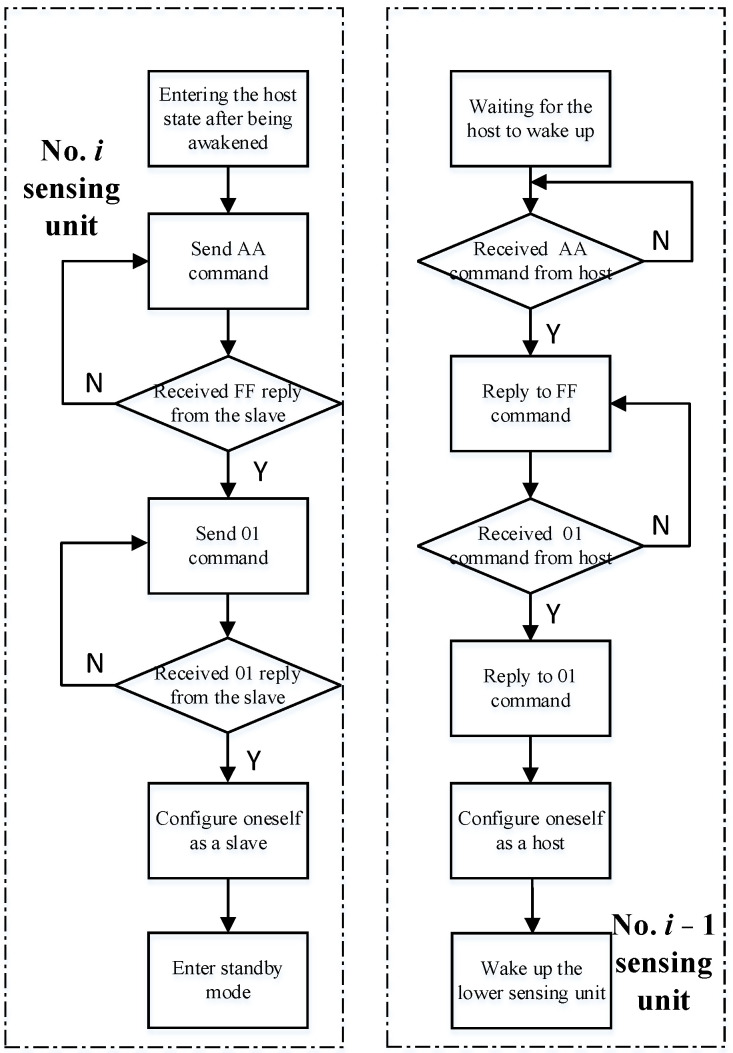
Program flowchart of the wake-up phase from top to bottom.

According to the overall workflow of the monitoring system, the software workflow of the sensing unit can be divided into two parts: the wake-up phase from top to bottom and the communication phase from bottom to top. To ensure the accuracy of communication, the communication process of the sensing unit adopts the modes of ask and answer interchangeably. When the host sends functional instructions, the slave will generate corresponding responses. The program flowchart of the wake-up phase from top to bottom is shown in Figure 7. 

Taking the No. *i* sensing unit and No. *i* − 1 sensing unit as examples, the wake-up process of these two sensing units can be roughly divided into four steps. The specific procedure is as follows:

Step 1: After the No. *i* sensing unit is awakened by the No. *i* + 1 sensing unit, it enters the host state and sends a wake-up command of AA to the No. *i* − 1 sensing unit. Then, it enters the command-receiving state and waits for the return command from the No. *i* − 1 sensing unit.

Step 2: After the No. *i* − 1 sensing unit is awakened, it will reply with an FF command and then enter the command receiving state, waiting for the No. *i* sensing unit to send the command.

Step 3: After receiving the return value from the No. *i* − 1 sensing unit, the No. *i* sensing unit sends a 01 command to the No. *i* − 1 sensing unit, controlling it to be in the wake-up phase from top to bottom, and then entering the command receiving state, waiting for the return command from the No. *i* − 1 sensing unit.

Step 4: After receiving the 01 command, the No. *i* − 1 sensing unit responds to the No. *i* − 1 sensing unit 01 command, indicating that it has entered the wake-up phase from top to bottom. Then, configure itself as the host and continue to wake up the No. *i* − 2 sensing unit. At the same time, after receiving the return value from the No. *i* − 1 sensing unit, the No. *i* sensing unit configures itself as a slave device and enters standby mode.

Repeat the above process until the bottommost sensing unit is awakened.

**Figure 8:**
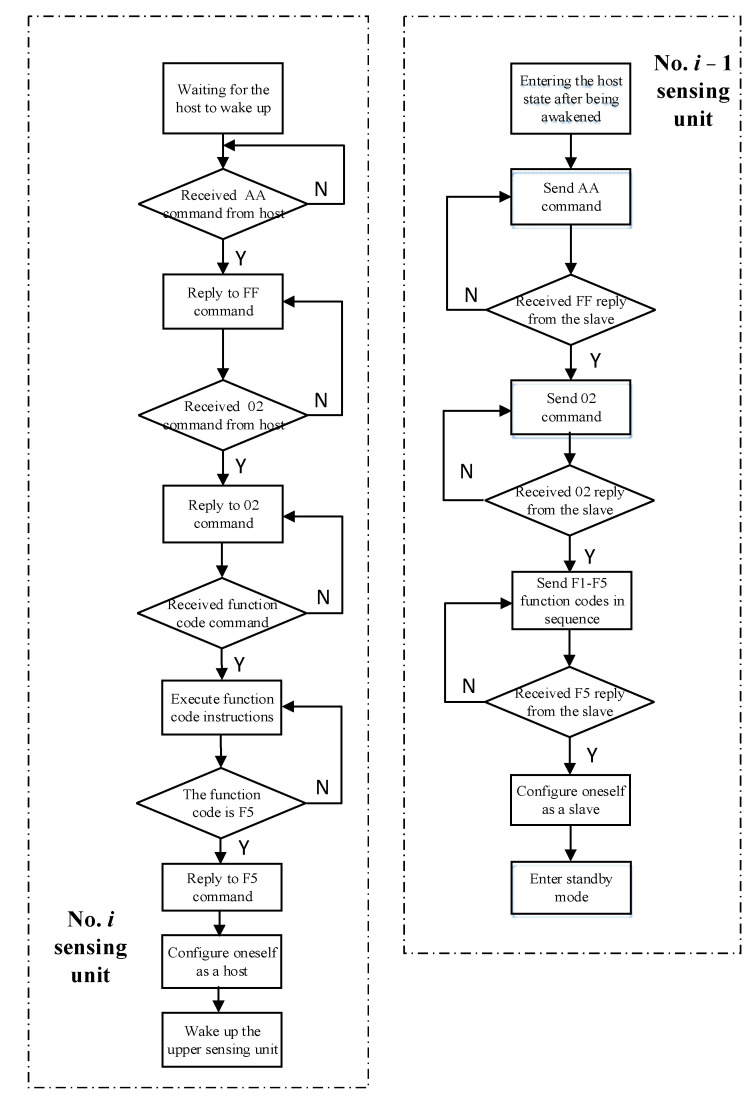
Program flowchart of the communication phase from bottom to top.

The program flowchart of the communication phase from bottom to top is shown in Figure 8. 

Taking the No. *i* sensing unit and the No. *i* − 1 sensing unit as examples, at this time, the No. *i* sensing unit serves as the slave and the No. *i* − 1 sensing unit serves as the host. The communication process between these two sensing units can be roughly divided into six steps. The specific content is as follows:

Step 1: The No. *i* − 1 sensing unit sends a wake-up command of AA to the No. *i* sensing unit and then enters the command receiving state, waiting for the return command from the No. *i* sensing unit.

Step 2: After the No. *i* sensing unit is awakened, it will reply to the FF command and then enter the command receiving state, waiting for the No. *i* − 1 sensing unit to send the command.

Step 3: After receiving the return value from the No. *i* sensing unit, the No. *i* − 1 sensing unit sends the 02 command to the No. *i* sensing unit, controlling it to be in the communication phase from bottom to top, and then enters the command-receiving state, waiting for the return command from the No. *i* sensing unit.

Step 4: After receiving the 02 command, the No. *i* sensing unit responds to the No. *i* − 1 sensing unit 02 command, indicating that it has entered the communication phase from bottom to top. Then enter the command-receiving state and wait for the No. *i* − 1 sensing unit to send the measurement command.

Step 5: The No. *i* − 1 sensing unit sends F1-F5 function code instructions to the No. *i* sensing unit in sequence. At the same time, the No. *i* sensing unit executes the corresponding functions in sequence after receiving the function code instructions from the No. *i* − 1 sensing unit.

Step 6: When the No. *i* sensing unit receives the F5 command sent by the No. *i* − 1 sensing unit, it responds with the F5 command from the No. *i* − 1 sensing unit and then configures itself as the host to continue to wake up the No. *i* + 1 sensing unit. At the same time, after receiving the F5 command replied to by the No. *i* sensing unit, the No. *i* – 1 sensing unit configures itself as a slave state and enters standby mode.

Repeat the above process until the top sensing unit completes the measurement, and then send the data to the ground management terminal for processing.

## 5. Experimental Testing

### 5.1. Sensing Array Underground Communication Experiment

**Figure 9:**
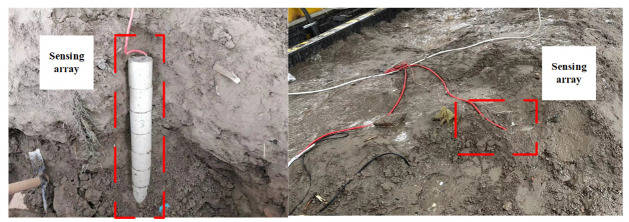
Underground wireless communication experiment with a sensing array.

Based on the experience of our research group in the on-site installation of underground displacement three-dimensional monitoring systems, after the installation of the monitoring system is completed, the sensing array will be in a mud–water mixture. The sensing units in the monitoring system transmit information through electromagnetic waves, and the electromagnetic wave signal will have a certain attenuation in the soil. Consequently, it is necessary to verify the normal communication capabilities of the sensing array in this environment. Embed the sensing array into the soil for communication experiments, as shown in Figure 9. 

**Figure 10:**
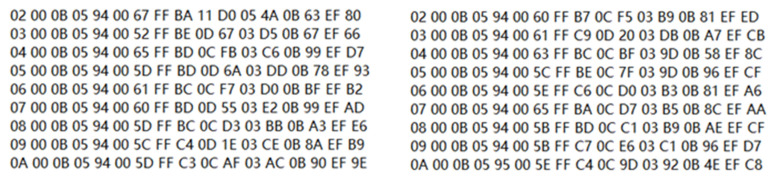
Underground wireless communication results.

After powering on the sensing array, the development board is used to receive data and display them on the upper computer, as shown in Figure 10. It can be seen that the data reception is corrected.

**Figure 11:**
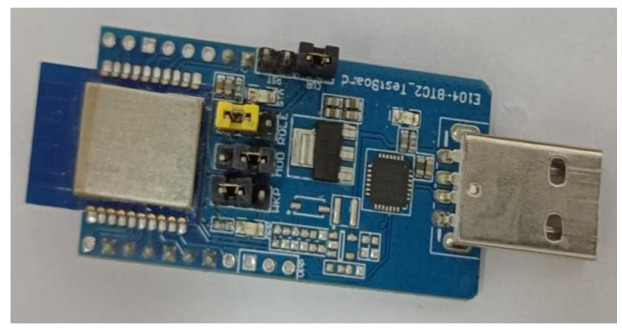
E104-BT02 Bluetooth test module.

**Figure 12:**
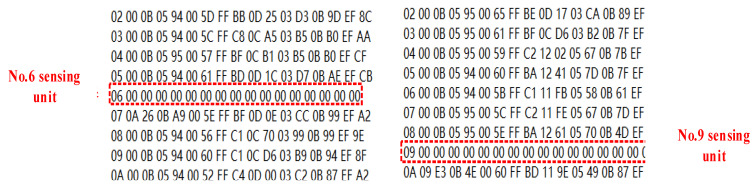
Communication results for simulating a sensing unit fault.

Subsequently, employ the E104-BT02 Bluetooth test module, as shown in Figure 11, to awaken the 6th or 9th sensing unit and control it to enter test mode. This simulates the scenario in which the No. 6 or No. 9 sensing unit malfunctions while operating in the field. The remaining sensing units continue to function normally, as shown in Figure 12.

Therefore, this experiment demonstrates that even when individual sensing units in the sensing array encounter issues, the remaining sensing units can still communicate normally.

### 5.2. Establishment of a Power Consumption Model for Sensing Array

**Figure 13:**
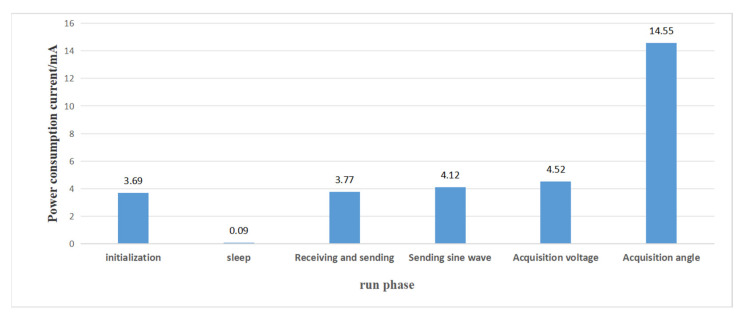
Actual measurement of the power consumption current.

The power consumption characteristics of the sensing unit in this monitoring system are extremely complex, and it is necessary to establish a power consumption model to evaluate the power consumption of the sensing array. According to the power consumption model, the number of sensing units and power supply in the sensing array can be adjusted to adapt to the field working environment. According to the working status of the sensing unit and the design of the circuit, the operation stages of the sensing unit are divided into six stages: initialization, receiving and sending, sending sine waves, acquisition voltage, acquisition angle, and sleep. By measuring with a multimeter, the consumed current at each operating stage is shown in Figure 13.

The initialization stage of the sensing unit is only at the moment of initial power on, which accounts for a very low proportion of the total power consumption of the sensing unit; therefore, the power consumption during the initialization phase can be ignored. Assuming the working cycle of the sensing array is *T*, the sensing array will remain in a sleep state for the rest of the time after completing communication within one cycle. According to the mode of ask and answer interchangeably designed by the software, it can be known that the functional modules of the sensing unit in the sending and receiving stages are the same, so their power consumption current is also consistent.

According to the software design, the operation of the sensing array can be divided into three parts: top-to-bottom wake-up mode, bottom-to-top communication mode, and standby mode. The top-to-bottom wake-up mode includes two operating states: receiving and sending, and sleep. According to the setting of the sensing unit program and actual testing, when the sensing unit is in the top-to-bottom wake-up mode, the time *T*_up_ of the sensing unit in both the host and slave states is 2 s. Assuming a sensing array composed of n sensing units with a power supply voltage of 7.5 V, the power consumption during the top-to-bottom wake-up mode is shown in Equation (3).

(3) $$\begin{array}{l} E_{\mathrm{up}-\mathrm{down}}=7.5\times (2\times 3.77\times T_{up}+0.09\times (n-2)\times T_{up})\times (n-1)\\ =1.35n^{2}+109.05n-111.4(mw\cdot s) \end{array}$$

In the bottom-to-top communication mode, the sensing unit in the host state includes four stages: sleep, data sending, sending sine waves, and angle acquisition. The sensing unit in the slave state includes three stages: sleep, data reception, and voltage acquisition. According to the setting of the sensing unit program and actual testing, it can be concluded that the time *T*_js_ required for the sensing unit to complete receiving and saving a piece of sensing unit data is about 0.4 s, the time *T*_sin_ of sending a sine wave is about 1 s, the time *T*_v_ required to acquire voltage is about 1 s, the time *T*_a_ required to acquire angle is about 0.6 s, and the rest of the time is the sleep time *T*_sleep_. 

When relay communication is carried out from bottom to top, the host state duration of the sensing unit is the same as the slave state duration. As the number of sensing units increases, the time of receiving and saving a piece of sensing unit data will also increase. According to the test, the time *t*_2_ of the No. 2 sensing unit being in the slave state is about 3.6 s, and the time *t*_k_ of the remaining sensing units in the slave state increases based on the No. 2 sensing unit, so *t*_k_ can be shown in Equation (4).

(4) $$t_{k}=t_{2}+(k-2)t_{js}=0.4k+2.8(s)$$

According to the workflow of the program design, when the sensing array operates for one cycle, the total power consumption of the sensing units in the slave state is shown in Equation (5).

(5) $$E_{\mathrm{slave}}=\sum_{k=2}^{n}(7.5\times t_{k}\times 3.77)+(7.5\times T_{v}\times 4.52)\times (n-1)\;(mw\cdot s)$$

The total power consumption of the sensing units in the host state is shown in Equation (6).

(6) $$E_{\mathrm{host}}=\sum_{k=2}^{n}(7.5\times t_{k}\times 3.77)+(7.5\times T_{\sin}\times 4.12+7.5\times T_{a}\times 14.55)\times (n-1)\;(mw\cdot s)$$

When the sensing array is in bottom-to-top communication mode, there are only two sensing units in working mode, and the remaining sensing units are in sleep mode. Therefore, the total power consumption of the other sensing units is shown in Equation (7).

(7) $$E_{\mathrm{other}}=\sum_{k=2}^{n}(7.5\times t_{k}\times 0.09)+7.5\times 0.09\times (T_{\sin}+T_{v}+T_{a})\times (n-1)\times (n-2)\;(mw\cdot s)$$

The No. 1 sensing unit occupies a special position in the entire sensing array. Due to its position at the lowest end of the entire sensing array, after completing the slave state of the top-to-bottom wake-up mode, the No. 1 sensing unit enters the host state of the bottom-to-top communication mode after a 3 s timer. Therefore, the power consumption of the No. 1 sensing unit is as shown in Equation (8).

(8) $$E_{1}=7.5\times (3.77\times (T_{\mathrm{up}}+3)+4.12\times T_{\sin}+14.55\times T_{a})=237.75\;(mw\cdot s)$$

The total power consumption of the bottom-to-top communication mode is shown in Equation (9).

(9) $$\begin{array}{l} E_{\mathrm{down}-\mathrm{up}}=E_{\mathrm{slave}}+E_{\mathrm{host}}+E_{\mathrm{other}}+E_{1}\\ =57.225\times \sum_{k=2}^{n}\left(0.4k+2.8\right)+1.755n^{2}+125.01n+110.985\;(mw\cdot s) \end{array}$$

In the remaining time, the sensing array is in standby mode, and all sensing units are in sleep mode. The standby mode time is shown in Equation (10).

(10) $$T_{\mathrm{standby}}=T-T_{\mathrm{up}}\times (n-1)-\sum_{k=2}^{n}(0.4k+2.8)-T_{v}\times (n-1)-T_{\sin}\times (n-1)-T_{a}\times (n-1)(s)$$

The total power consumption in standby mode is shown in Equation (11).

(11) $$\begin{array}{l} E_{\mathrm{sleep}}=(7.5\times 0.09\times T_{\mathrm{standby}})\times n\\ =-0.675n\times \sum_{k=2}^{n}\left(0.4k+2.8\right)-3.105n^{2}+(0.675T+3.105)n\;(mw\cdot s) \end{array}$$

The total power consumption of the improved monitoring system in one cycle is shown in Equation (12).

(12) $$\begin{array}{l} E_{\mathrm{new}}=E_{\mathrm{up}-\mathrm{down}}+E_{\mathrm{down}-\mathrm{up}}+E_{\mathrm{standby}}\\ =(57.225-0.675n)\times \sum_{k=2}^{n}\left(0.4k+2.8\right)+(237.165+0.675T)n-0.415\;(mw\cdot s) \end{array}$$

Under a power supply voltage of 7.5 V, the average working current of a single sensing unit in the original monitoring system is measured to be 22 mA. Assuming that the sensing array works for *T*_old_ once, the power consumption of the sensing array composed of *n* sensing units working once is shown in Equation (13):

(13) $$E_{\mathrm{old}}=7.5\times 22\times n\times T_{\mathrm{old}}=165nT_{\mathrm{old}}\;(mw\cdot s)$$

For example, the number of sensing units in a sensing array is one hundred, and the sensing array collects data every half hour. Substituting *n* = 100 and *T* = *T*_old_ = 1800 into Equations (12) and (13), respectively, the power consumption of the improved system and the original system can be obtained as shown in Equations (14) and (15), respectively.

(14) $$E_{new}\approx 121616.465\;(mw\cdot s)\approx 33.78\;(mw\cdot h)$$

(15) $$E_{old}=29700000\;(mw\cdot s)=8250\;(mw\cdot h)$$

According to Equation (16), the improved system power consumption is only 0.409% of the original system power consumption.

(16) $$\frac{E_{new}}{E_{old}}=\frac{33.78}{8250}\times 100\%\approx 0.409\%$$

### 5.3. Measurement of the Sensing Array Power Consumption

**Figure 14:**
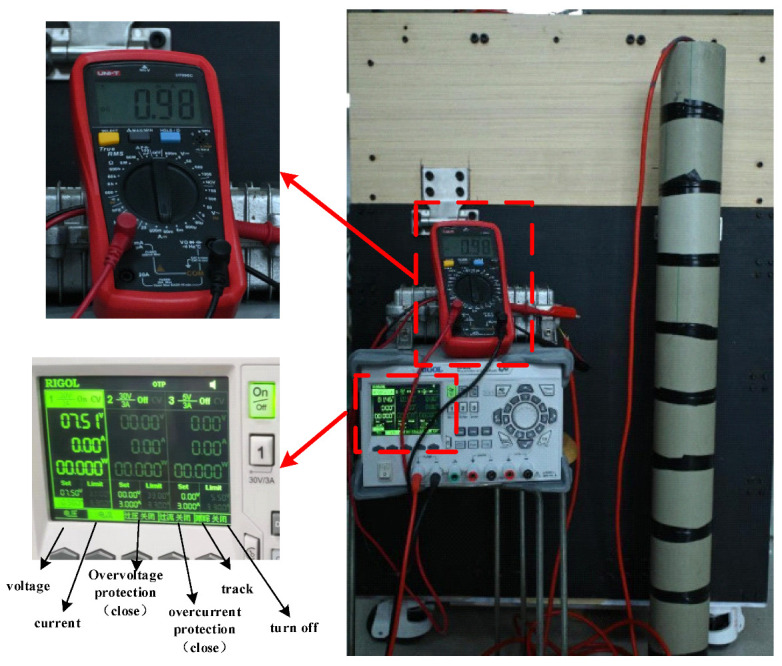
Sensing array power consumption test.

In order to verify the accuracy of the power consumption model, 10 sensing units are selected to compose a sensing array (i.e., *N* = 10), and the power consumption of the sensing array is experimentally tested. Assuming data are collected every half hour, and due to the limitations of experimental conditions, a multimeter current range is used instead of a transient current measurement instrument in this experiment. The multimeter is connected to the power line between the sensing array and the ground management terminal, and the current value of the multimeter is recorded. The power consumption of the entire operating state of the sensing array can be obtained, as shown in Figure 14.

**Figure 15:**
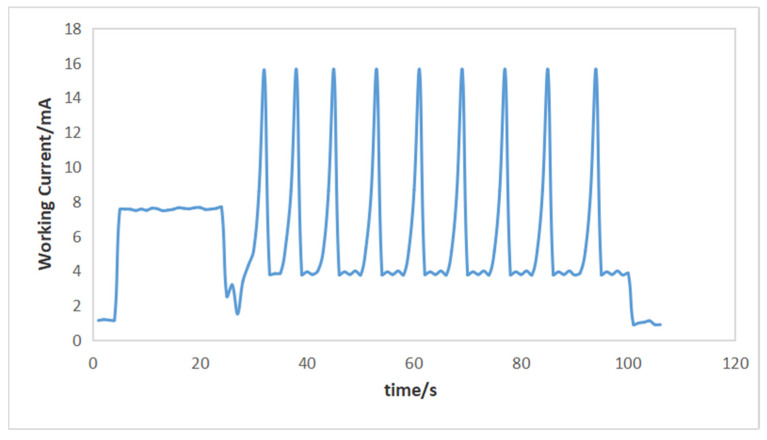
Current variation of the sensing array operating state.

By recording the changes in the current value of the multimeter and extracting the current value per second, the obtained results are shown in Figure 15.

By analyzing Figure 15, it can be observed that the current *I*_sleep_ consumed by the sensing array in standby mode is about 1.02 mA. The average current *I*_U-D_ consumed during top-to-bottom wake-up mode is about 7.36 mA, and the time *T*_U-D_ is about 25 s. When the sensing array enters the bottom-to-top communication mode, it needs to be calculated separately because there are three different stages: the voltage value acquisition stage, the attitude information acquisition stage, and the data transmission stage. The working current *I*_a_ in the attitude information acquisition stage is about 15.1 mA, and the time *T*_a_ is about 9 s. The average working current *I*_st_ during the voltage value acquisition stage and data transmission stages is about 3.77 mA, and the time *T*_st_ is about 61 s. The sensing array undergoes measurement work every half an hour and remains in standby mode for the rest of the time. Considering the power supply voltage of 7.5 V, the required energy consumption is shown in Equation (17).

(17) $$\begin{array}{l} E=7.5\times (I_{U-D}\times T_{U-D}+I_{a}\times T_{a}+I_{\mathrm{st}}\times T_{\mathrm{st}}+I_{\mathrm{sleep}}\times (1800-T_{U-D}-T_{a}-T_{\mathrm{st}}))\\ \approx 17167.275\;(mw\cdot s)\approx 4.77\;(mw\cdot h) \end{array}$$

By substituting *n* = 10 and *T* = 1800 into Equation (11), the energy consumption is calculated as shown in Equation (18).

(18) $$E_{n=10}\approx 16883.465\;(mw\cdot s)\approx 4.69\;(mw\cdot h)$$

According to Equation (18), the error between the calculated value of the established power consumption model and the experimental measurement value is only 1.71%. The error indicates that this power consumption model can effectively describe the power consumption of the sensing array; therefore, the power consumption model can provide a reference for the subsequent installation of the monitoring system in the field.

(19) $$\left|E-E_{N=10}\right|/E_{N=10}\times 100\%=\left|4.77-4.69\right|/4.69\times 100\%\approx 1.71\%$$

### 5.4. Sensor Displacement Measurement Experiment

**Figure 16:**
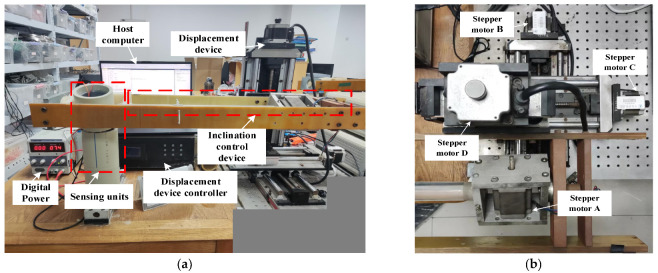
Displacement experimental platform. (**a**) Composition of the platform; (**b**) stepping motors.

The experimental platform for the displacement measurement experiment is shown in Figure 16. The experimental platform mainly consists of a digital power supply, a host computer, two sensing units, a displacement device controller, a displacement device, and an inclination control device. Among them, the digital power supply supplies power to two sensing units. The sensing unit located at the lower relative position serves as the excitation end, while the other sensing unit serves as the measurement end. The excitation end is fixed on a smooth horizontal plane, and the measurement end is fixed on the rotating arm of the inclination control device. The inclination control device consists of the stepper motor A and a rotating arm. By controlling the stepper motor A to drive the rotating arm to rotate, the tilt angle of the measurement end relative to the excitation end is controlled (*θ)*. The displacement control device consists of stepper motor B, stepper motor C, and stepper motor D. By controlling the three stepper motors through the displacement device controller, the rotating arm can move in the X, Y, and Z directions, thereby controlling the relative horizontal displacement *r* and relative vertical displacement *z* of the measurement end relative to the excitation end. The measurement range of horizontal displacement and vertical displacement is 0–50 mm, and the change step is 1 mm. The variation range of tilt angle is 0°–80°, and the variation interval is 5°.

**Figure 17:**
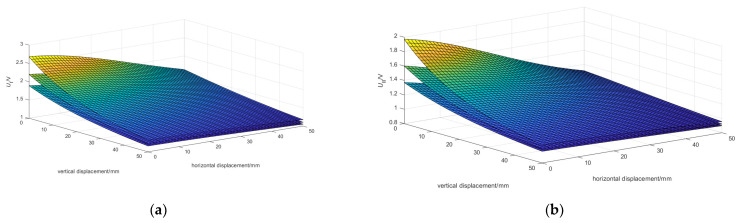
Three-dimensional surface plots of double mutual inductance voltage vs. r and z. (**a**) Three-dimensional surface plot of type I mutual inductance voltage *U*_1_ vs. *r* and *z*; (**b**) Three-dimensional surface plot of type II mutual inductance voltage *U*_2_ vs. *r* and *z*.

Through experiments, fix the tilt angle *θ* between the excitation end and the measurement end, change the value of the relative horizontal displacement *r* and the relative vertical displacement *z*, and collect a dataset of the type I mutual inductance voltage *U*_1_ and the type II mutual inductance voltage *U*_2_. Then, change the tilt angle *θ* and collect multiple sets of data. Figure 17a,b are three-dimensional surface plots depicting the relationship among the relative horizontal displacement *r*, relative vertical displacement *z*, and the type I mutual inductance voltage *U*_1_ and type II mutual inductance voltage *U*_2_, respectively, at different tilt angles, which are 15°, 30°, and 45° from top to bottom.

**Figure 18:**
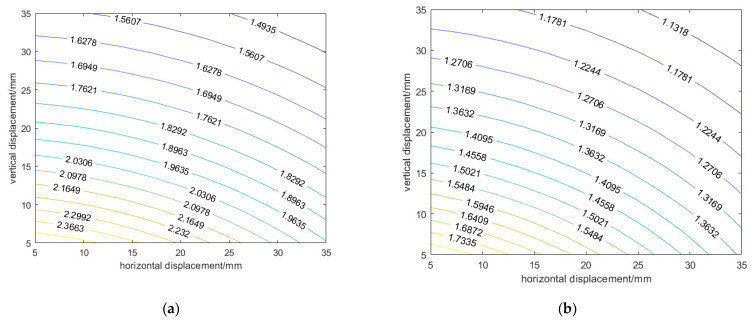
Dual inductance voltage contour lines. (**a**) Type I mutual inductance voltage *U*_1_ contour lines; (**b**) Type II mutual inductance voltage *U*_2_ contour lines.

By analyzing Figure 17, it can be seen that when the relative horizontal displacement *r* and relative vertical displacement *z* remain constant, the type I mutual inductance voltage *U*_1_ and the type II mutual inductance voltage *U*_2_ will decrease with the tilt angle *θ* increasing. Taking the tilt angle *θ* equal to 15° as an example, connect the points where the type I mutual inductance voltage *U*_1_ is equal as r and z change. The contour lines of the type I mutual inductance voltage *U*_1_ can be obtained, as shown in Figure 18a. Similarly, the contour lines of the type II mutual inductance voltage *U*_2_ can be obtained, as shown in Figure 18b.

**Figure 19:**
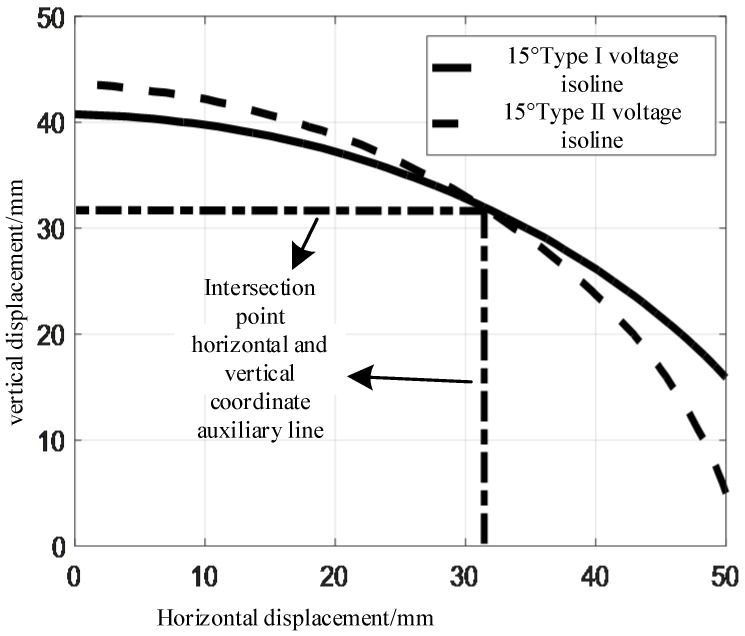
Intersection of double mutual inductance equivalent voltage lines.

Observing Figure 17 and Figure 18, it can be seen that when the tilt angle *θ* remains constant and the relative horizontal displacement *r* and relative vertical displacement *z* monotonically increase, type I mutual inductance voltage *U*_1_ and type II mutual inductance voltage *U*_2_ monotonically decrease. When the relative horizontal displacement *r* and relative vertical displacement *z* are constant, the combination of the corresponding type I mutual inductance voltage *U*_1_ and type II mutual inductance voltage *U*_2_ is unique. At this tilt angle *θ*, the relative horizontal displacement *r* and relative vertical displacement *z* can be represented by the combination of the type I mutual inductance voltage *U*_1_ and the type II mutual inductance voltage *U*_2_, as shown in Figure 19.

Observing Figure 19, it can be seen that at the tilt angle of 15°, there is an intersection point between a type I mutual inductance voltage *U*_1_ contour line and a type II mutual inductance voltage *U*_2_ contour line. The relative horizontal displacement *r* and the relative vertical displacement *z* corresponding to this intersection point are the relative displacements between the measurement end and the excitation end. The detailed calculation process can be found in the literature [12].

The displacement measurement of the sensor was carried out on the experimental platform in Figure 16. Two different tilt angles of 16.45° and 35.65° are randomly selected. The comparison between the measured values of the sensor and the actual displacement values of the experimental platform is shown in Table 7. It can be seen that the measurement error of the sensor is less than 1 mm.

## 6. Conclusions

The underground displacement three-dimensional monitoring system is usually installed in unmanned outdoor fields, so the stability and endurance of the overall system are very important. In order to ensure the long-term stable operation of the monitoring system, this article redesigns the underground displacement three-dimensional monitoring system to address the relevant issues of the original system. Compared to the original system, this monitoring system has the following characteristics:

1. Redesign the monitoring system using low-power methods and establish a power consumption model. The original system did not fully consider low-power design in its design, resulting in the system only being able to operate for less than a day in rainy and cloudy environments. Therefore, this article adopts low-power design methods and techniques to redesign this monitoring system. In terms of hardware, low-power components are selected, the sensing unit circuit is modularized, and controllable power management is adopted. In terms of software, multiple working modes are divided and combined with controllable power management modules to reasonably arrange the working time of each functional module of the sensing unit, achieving dynamic power management of the sensing unit. Ultimately, the overall power consumption of the monitoring system was reduced. By establishing a power consumption model, it is found that when the number of sensing units in the improved system is 100, the power consumption of the entire sensing array is only 0.409% of the original system, greatly reducing the power consumption of the underground displacement three-dimensional monitoring system and extending the system’s usage time. By comparing the actual measurement results with the calculated values of the power consumption model, it can be found that when there are 10 sensing units, the error is only 1.71%. It can provide a reference for the subsequent selection of the number of sensing units to be installed in the field and the selection of power supply parameters.
2. The wired communication in the monitoring system has been upgraded to Bluetooth wireless communication. The original system used the RS485 bus to connect the ground management terminal and hundreds of sensing units for communication. If any sensing unit 485 communication module fails, it will cause other sensing units to be unable to continue communication. This article addresses this issue by adopting Bluetooth wireless communication instead of wired communication. A relay wireless communication network suitable for this monitoring system is designed based on the BLE network topology structure, dividing the sensing array into three working modes to avoid the problem of the entire sensing array being scrapped and unusable due to the failure of a single sensing unit.
3. The measurement accuracy has not decreased. While changing the communication mode and employing a low-power design of the underground displacement three-dimensional monitoring system, the experimental results show that when the horizontal displacement and vertical displacement are within the measurement range of 0–50 mm, the maximum measurement error of this method will not exceed 1 mm, meeting the needs of actual underground displacement measurement.
4. Future direction: In the future, optimization can be pursued in two aspects. Firstly, there is still a power line between sensing units, making the processing, transportation, and installation of the sensing units require careful consideration. Subsequently, it may be worth considering using wireless charging between sensing units for power supply. Secondly, the high sensitivity and accuracy of the underground displacement three-dimensional monitoring system enable us to detect early signs of underground movement, providing more comprehensive data support for disaster risk assessment and management. By collecting data from this monitoring system, a more effective and accurate geological hazard prediction model can be established to predict geological hazards such as landslides and debris flows.

## Figures and Tables

**Table 1:** Comparison of underground displacement detection methods.

References	Underground Displacement Monitoring Methods	Advantage	Disadvantage
[6,9]	borehole inclinometer	Low cost and simple structure	Low degree of automation, only able to measure horizontal displacement
[5,8]	optical fiber sensing technology	Flexible deployment and a high degree of automation	Easy to fracture under large deformation conditions
[7]	array displacement meter	Waterproof, corrosion-resistant, and strong anti-interference ability	Due to its structure, the maximum bending angle of each subarray segment is 60°
[12]	Double mutual inductance measurement method	Waterproof, achieving three-dimensional displacement measurement	High power consumption and damage to a single sensing unit can easily lead to sensor failure

**Table 2:** Comparison of short-distance wireless communication technologies.

References	Communication Technology	Communication Distance	Consumption	Advantage	Disadvantage
[31]	infraredcommunicat	About 10 M	A few milliwatts to tens of milliwatts	Simple structure and low cost	Easy to be affected by obstructions, point-to-point connection
[24,26]	Zigbee	50–300 M	**Standby power consumption:** several microwatts **Transmission power consumption:** several milliwatts	Low power consumption, supporting a large number of device connections	Low transmission speed and susceptibility to interference from walls and other obstacles
[32]	WiFi	100–300 M	Several tens of milliwatts	High-speed data transmission, Wide coverage range	High power consumption, Device connection restricted Larger size
[27,28,29,30]	BLE	10–100 M	**Standby power consumption:** several microwatts **Transmission power consumption:** several milliwatts	Low power consumption, Multi-device connection	Short transmission distance and relatively low transmission rate

**Table 3:** Current of STM32L151C8T6 in various operating modes.

Working Mode	Normal Operation	Sleep Mode	Shutdown Mode	Standby Mode
Working current	36 mA	14.4 mA	14 μA	2.8 μA

**Table 4:** Main parameter table of the functional modules.

Functional Modules	Main Chips	Supply Voltage Range	Operating Current	Other Parameters
MCU	STM32L151	3.3 V	Run mode: 185 μA/Hz Standby mode: 0.28 μA	Basic Frequency: 32 MHz RAM: 16 K FLASH: 256 K
Attitude detection module	JY901S	3.3 V~5 V	<25 mA	Accuracy: 0.01° Angle range: −180°~180°
Mutual inductance voltage detection circuit	TLV9052	5 V	330 μA/channel	Slew rate: 15 V/μs Offset voltage: ±0.5 μV/°C Voltage noise density: 15 nV/√Hz
Communication module	E104BT-02	2.5 V~3.6 V	Emission mode: 3.4 mA Receiving mode: 3.7 mA Sleep mode: 13 μA	Working Frequency: 2.4 GHz BAUD: 19200 receiver sensitivity: −96 dBm

**Table 5:** Communication protocol for the function codes.

Serial Number	1	2	3	4
Format sequence	First data	Data length	function code	check code
Data definition	0xAA/0xFF	0x05	/	CRC32check code
Bytes occupied	1 byte	1 byte	1 byte	2 bytes

**Table 6:** Function codes.

Function Codes	Function	Function Codes	Function
0xAA	Host wake-up slave command	0xF1	Collect the value of *U*_1_
0xFF	Reply to the host after the slave is awakened by the host	0xF2	Collect the value of *U*_2_
0x01	Relay direction from top to bottom	0xF3	Read attitude detection module data
0x02	Relay direction from bottom to top	0xF4	Store the data of the sensing unit below
0xF0	Start the measurement preparation work	0xF5	Measurement work is over

**Table 7:** Comparison of sensor displacement measurements and actual displacement values (unit: mm).

Actual Displacement/mm (r, z)	Measuring Displacement/mm	
	*θ* = 16.45°	*θ* = 35.65°
(5, 5)	(5.07, 4.47)	(5.03, 4.81)
(10, 10)	(9.93, 9.6)	(9.94, 9.75)
(15, 15)	(14.85, 14.71)	(15.03, 14.90)
(20, 20)	(19.85, 19.70)	(20.00, 20.20)
(25, 25)	(24.79, 24.72)	(25.11, 24.97)
(30, 30)	(29.72, 29.70)	(30.06, 29.89)
(35, 35)	(34.85, 34.75)	(35.24, 34.90)
(40, 40)	(39.97, 39.87)	(40.38, 40.08)
(45, 45)	(44.65, 44.90)	(45.23, 45.13)
maximum error	(−0.35,−0.53)	(0.38,−0.25)
variance	(0.0162,0.0167)	(0.0201,0.0225)

## Data Availability

The data presented in this study are available on request from the corresponding author.
